# *The Organon* and Materia Medica Club of the Bay Cities of California

**Published:** 1897-04

**Authors:** W. E. Ledyard

**Affiliations:** B. A., M. B., M. R. C. S. Eng., Secretary


### *THE ORGANON* AND MATERIA MEDICA CLUB OF THE BAY CITIES OF CALIFORNIA.

The regular semi-monthly meeting was held at the office of Dr. George H. Martin, 606 Sutter Street, San Francisco, Friday evening, November 20th, 1896.

The meeting was called to order by the President, Dr. J. M. Selfridge, at 8 P. M.

Members present: Drs. J. M. and C. M. Selfridge, M. T. Wilson, and W. E. Ledyard.

The reading of the minutes of the previous meeting was dispensed with, in consequence of the absence of the Secretary.

*The Organon* was read, Sections 223 to 230, inclusive.

### Discussion.

Dr. J. M. Selfridge—To illustrate the fact that insanity is apt to follow brain lesion, gave the case of a man who had lost all moral sense and had become, not only lascivious, but murderous. On the removal of the calvarium, those parts of the brain which correspond phrenologically to the moral qualities were disorganized, while those supposed to control combativeness and destructiveness were inflamed and congested with blood.

In a second case, to illustrate the effect of shock, two men were out hunting. One was shot through the tibia, the accident being followed by *inability to pass water, from the shock*, which *Hypericum* 1x overcame.

Dr. Wilson reported a case of nervous prostration cured by *mesmeric influence.*

Dr. J. M. Selfridge reported having made a successful use of the *mesmeric power,* in the case of a fall on the back of the head.

Dr. Ledyard mentioned a case of labor, in which the pains were very much relieved by passes made over the hips; also the case of a child where heart disease and Bright's disease of the kidneys were presumably caused by fear.

Dr. Wilson alluded to the fact that Dr. Campbell, of Southern California, lately advocated *kind treatment of the insane,* so long ago emphasized by Hahnemann.

The Club adjourned to meet at the same place and hour, on the first Friday in December.

W. E. LEDYARD, B. A., M. B., M. R. C. S. Eng.,
*Secretary.*

---

